# Estimation of plant height and yield based on UAV imagery in faba bean (*Vicia faba* L.)

**DOI:** 10.1186/s13007-022-00861-7

**Published:** 2022-03-05

**Authors:** Yishan Ji, Zhen Chen, Qian Cheng, Rong Liu, Mengwei Li, Xin Yan, Guan Li, Dong Wang, Li Fu, Yu Ma, Xiuliang Jin, Xuxiao Zong, Tao Yang

**Affiliations:** 1 National Key Facility for Crop Gene Resources and Genetic Improvement/Institute of Crop Sciences, Chinese Academy of Agricultural Sciences, Haidian District, Beijing, 100081 China; 2 Institute of Farmland Irrigation, Chinese Academy of Agricultural Sciences, Xinxiang, 453002 China; 3 Department of Horticulture, Washington State University, Pullman, WA 99164 USA

**Keywords:** Faba bean (*Vicia faba* L.), Unmanned aerial vehicle (UAV), Plant height, Yield estimation, Machine learning

## Abstract

**Background:**

Faba bean is an important legume crop in the world. Plant height and yield are important traits for crop improvement. The traditional plant height and yield measurement are labor intensive and time consuming. Therefore, it is essential to estimate these two parameters rapidly and efficiently. The purpose of this study was to provide an alternative way to accurately identify and evaluate faba bean germplasm and breeding materials.

**Results:**

The results showed that 80% of the maximum plant height extracted from two-dimensional red–green–blue (2D-RGB) images had the best fitting degree with the ground measured values, with the coefficient of determination (R^2^), root-mean-square error (RMSE), and normalized root-mean-square error (NRMSE) were 0.9915, 1.4411 cm and 5.02%, respectively. In terms of yield estimation, support vector machines (SVM) showed the best performance (R^2^ = 0.7238, RMSE = 823.54 kg ha^−1^, NRMSE = 18.38%), followed by random forests (RF) and decision trees (DT).

**Conclusion:**

The results of this study indicated that it is feasible to monitor the plant height of faba bean during the whole growth period based on UAV imagery. Furthermore, the machine learning algorithms can estimate the yield of faba bean reasonably with the multiple time points data of plant height.

**Supplementary Information:**

The online version contains supplementary material available at 10.1186/s13007-022-00861-7.

## Background

Faba bean (*Vicia faba* L.) is a cool-season legume crop, planted worldwide as a protein source of food, livestock feed, vegetables and industrial raw materials [1, 2]. According to the FAO statistical data (FAOSTAT; http://www.fao.org/), there were 71 countries and regions in the world planting dry faba bean in 2019. The harvesting area was 2,577,201 square hectometers (hm^2^), and the total production reached 5,431,503 tons (t), while the harvesting area of China was 839,618 hm^2^ and the production was 1,740,945 t, respectively. Therefore, China is the most important producer globally [3]. Faba bean is rich in protein, carbohydrates, minerals and vitamins [4]. Especially, the protein content of dry faba bean ranges from 20.3 to 41%, with an average of 27.6%, which is higher than pea, mungbean, cowpea and other legumes [5].

Plant height is a vital growth indicator of crops, which is related to plant architecture, lodging resistance and yield performance [6–8]. Early yield estimation is crucial for agricultural practices and it could provide farmers with field management decisions such as fertilization, irrigation and pesticide application [9]. Using early-season data for yield estimation could not only reduce resource input and environmental pollution, but also increase crop yield and subsequent profits [10, 11]. Therefore, we need access to crop height and yield efficiently and non-destructively [12–14]. The traditional method for plant height measurement through measuring the vertical distance from the ground to the top of the main stem in the natural state by the ruler [15] is labor-consuming, low throughput, destructive and prone to errors in the sampling. Moreover, plant height obtained by sampling several single plants is insufficient to represent the variations of all plants within the field [16, 17].

With the development of unmanned aerial vehicle (UAV) platform and miniaturization of sensors, it provides an alternative approach for crop height measurement [18]. UAV remote sensing technology is based on the platform of UAV and different sensors, which has the advantages of convenient operation, high flexibility, strong adaptability and low cost, therefore it is booming in agriculture research community [19–21].

RGB camera [22, 23], multispectral or hyperspectral camera [24], lidar [25, 26] and ultrasonic sensors [27, 28] have been applied on the UAV platform. These high-throughput phenotyping platforms have been implemented in many crops such as maize [29, 30], wheat [31, 32], rice [33, 34], sorghum [35, 36], soybean [37] in terms of plant height study. However, there was no report for faba bean. In previous studies, Han et al. [38] used the high-resolution RGB camera based on UAV platform to access to the multi-temporal images of corn in the field. Then plant height data was extracted by three-dimensional reconstruction point cloud model and they found that the extracted plant height was highly correlated with that of manual measurement. Holman et al. [39] developed a rapid and accurate method for extracting plant height, and compared it to the standard field measurement (R^2^ ≥ 0.77, RMSE ≤ 0.07 m). Demir et al. [40] filtered a digital surface model to derive the plant height, and compared the results with field measurement (Mean = 4.66 cm, Median = 3.75 cm, Standard Deviation = 13.78 cm).

Various methods have been proposed and applied to crop yield estimation, such as crop growth models, remote sensing data, and crop growth models coupled with environmental factors. Jin et al. [41] developed a winter wheat yield estimation method by combining AquaCrop model with optical and radar imaging data using the position and orientation system algorithm, which resulted in highly correlations between predicted and measured yield. Tao et al. [42] estimated the yield of winter wheat by using three regression methods with variables of spectral indices, plant height extracted from UAV hyperspectral images and the ground-measured plant height. Gilliot et al. [43] showed the potential of predicting maize yield based on the plant height extracted from UAV imagery, which would be better for variability analysis in the field trials. Feng et al. [44] performed an in-season alfalfa yield estimation using an ensemble machine learning model, and the results demonstrated the efficacy of the proposed ensemble model. Sun et al. [45] developed six mainstream machine learning models to estimate the potato tuber yield and obtained satisfactory estimation results, which demonstrated the potential of combining hyperspectral imagery with machine learning in yield estimation.

These studies explored the application of different sensors to obtain plant height data. Few of them compared the accuracy of extracting plant height from two-dimensional red–green–blue (2D-RGB), two-dimensional multispectral (2D-MS) and three-dimensional red–green–blue (3D-RGB) images. Furthermore, to our knowledge, the detection of estimating the faba bean yield based on machine learning algorithms has not been investigated so far in the literatures. Therefore, the aims of the current study were to (1) evaluate the relationship between the ground measured plant height and digital features extracted from UAV imagery, (2) explore the correlation between plant height and yield of faba bean in different time points, (3) evaluate the accuracy of faba bean yield estimation based on plant height by using three machine learning algorithms (SVM, RF and DT).

## Results

### Results of plant height extracted from UAV imagery

**Fig. 1:**
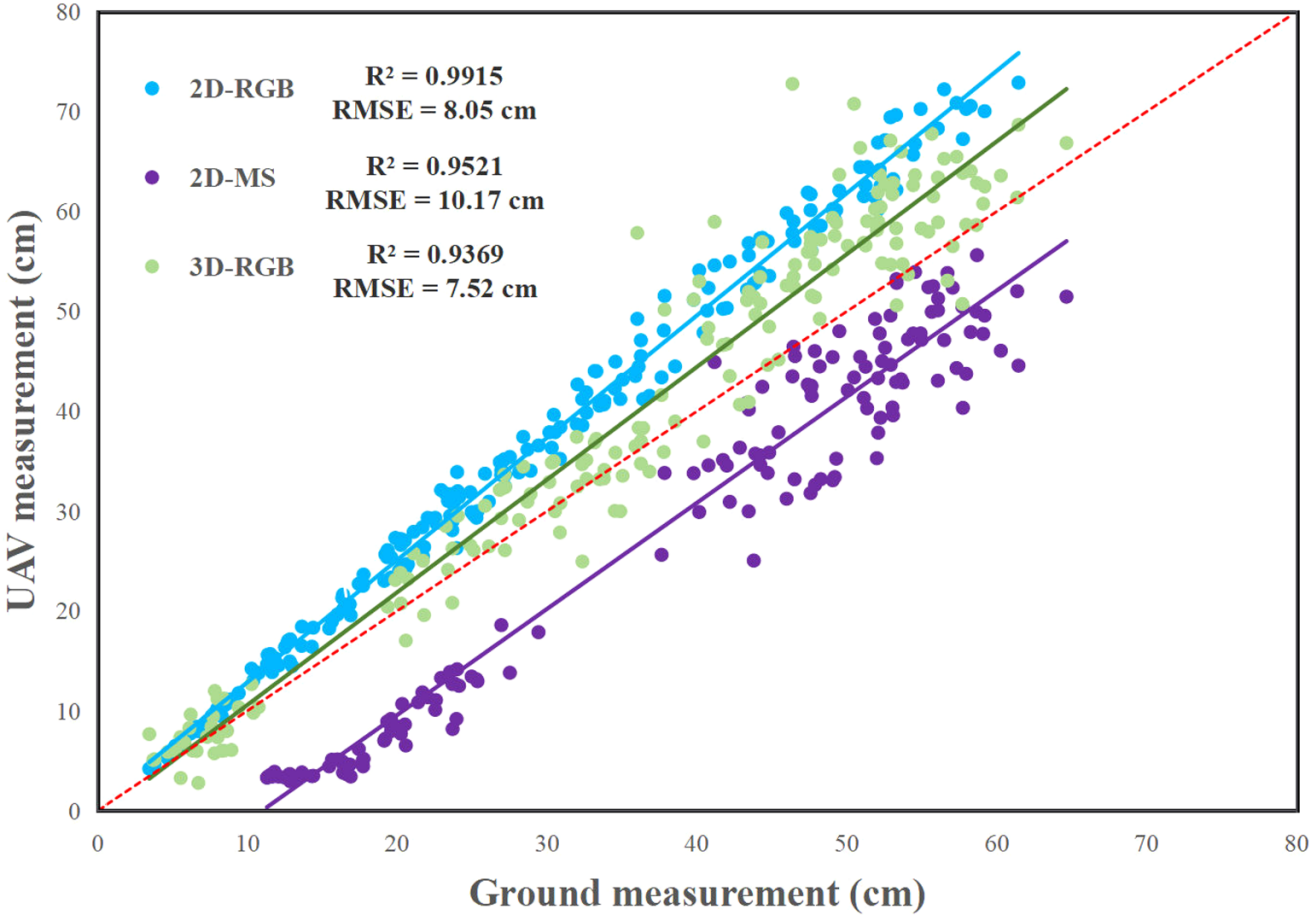
Results of extracting faba bean plant height from three types of UAV imagery

The faba bean plant height values extracted from three types of UAV imagery were compared with the ground measured values, and the results were shown in Fig. 1.

From Fig. 1, it showed that the plant height extracted based on 2D-RGB were all higher than the ground measured value (R^2^ = 0.9915, RMSE = 8.05 cm), and the plant height extracted based on 2D-MS were almost all lower than the ground measured value (R^2^ = 0.9521, RMSE = 10.17 cm), while the most of plant height extracted based on 3D-RGB were higher than the ground measured value, and a few were lower than it (R^2^ = 0.9369, RMSE = 7.52 cm). The R^2^ between the plant height extracted from these three types of UAV imagery and the ground measured values were all greater than 0.9, which showed that the plant height extracted from the UAV imagery had a strong correlation with the ground measured value, and this method can be used to measure plant height in *V. faba*.

**Fig. 2:**
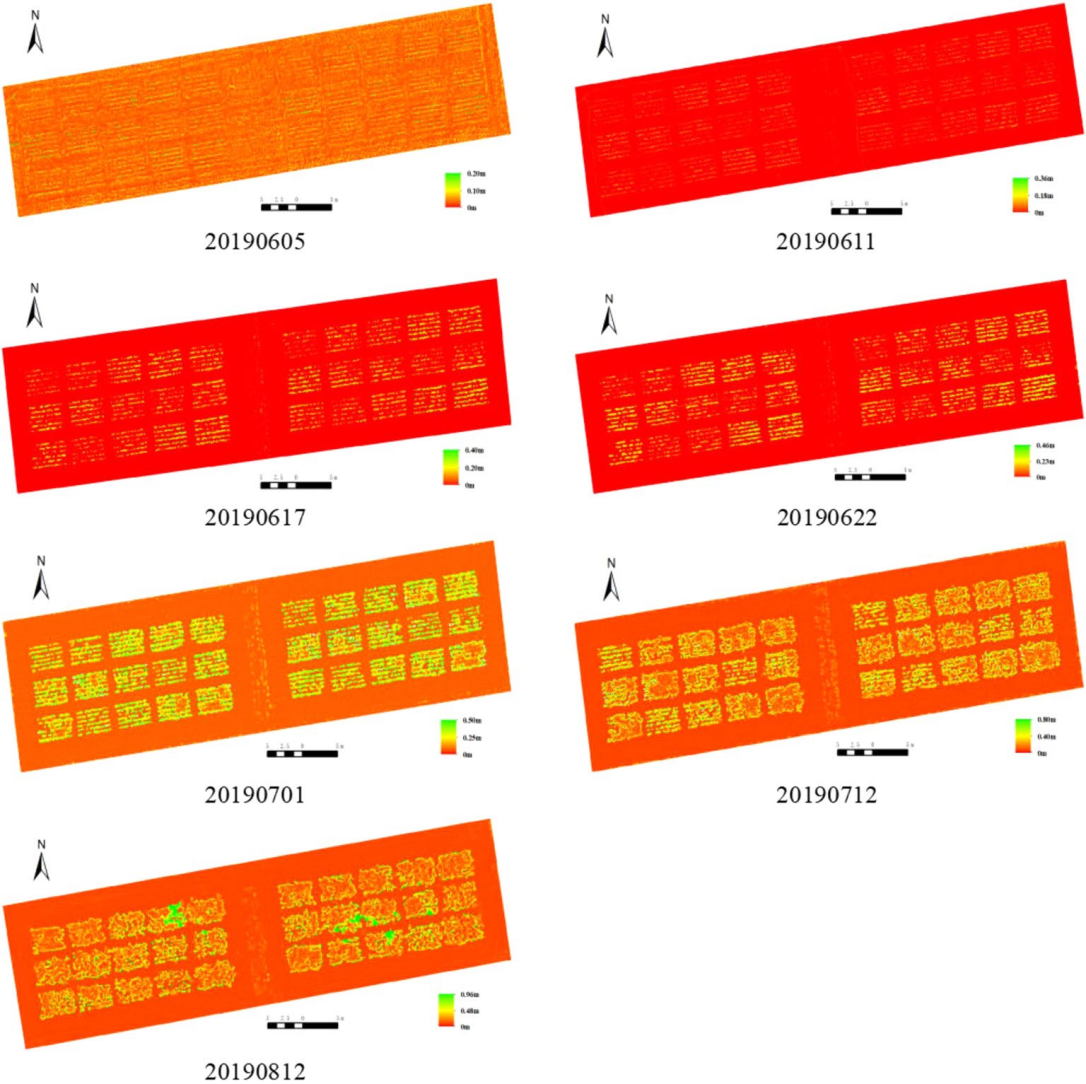
Spatial distribution of faba bean plant height in different time

The correlation of plant height between the ground measurement and extraction from 2D-RGB UAV imagery was the strongest among these three types of UAV imagery, and the spatial distribution map of faba bean plant height in different time were shown in Fig. 2.

### Calibration of plant height values extracted from 2D-RGB, 2D-MS and 3D-RGB

By comparing the plant height extracted from three types of UAV imagery with those measured on the ground, it was found that the maximum value of R^2^ was 2D-RGB (0.9915), followed by 2D-MS (0.952) and 3D-RGB (0.9399). The smallest value of RMSE was 3D-RGB (7.52 cm), followed by 2D-RGB (8.05 cm) and 2D-MS (10.17 cm). The smallest value of NRMSE was 3D-RGB (20.30%), followed by 2D-MS (26.97%) and 2D-RGB (28.05%). According to these evaluation indicators, it could be found that the extracted data with high coefficient of determination (R^2^) had relatively large errors (RMSE, NRMSE), while the data with small errors had relatively low coefficient of determination, so it was difficult to judge which type of UAV imagery was the most appropriate one for extracting the faba bean plant height in this study. Therefore, the plant height values extracted from three types of UAV imagery were calibrated in proportion of 70% ~ 100% (Table 1).

**Table 1:** Statistical results of plant height in different proportion

Type	PH%	R^2^	RMSE (cm)	NRMSE (%)	Fitting equation
2D-RGB	70%	0.9915	4.45	15.51	y = 0.8571x + 0.4299
	75%	0.9915	2.62	9.12	y = 0.9184x + 0.4606
	**80%**	**0.9915**	**1.44**	**5.02**	**y = 0.9796x + 0.4909**
	85%	0.9915	2.34	8.15	y = 1.0408x + 0.522
	90%	0.9915	4.13	14.39	y = 1.102x + 0.5527
	95%	0.9915	6.07	21.15	y = 1.1633x + 0.5834
	100%	0.9915	8.05	28.05	y = 1.2245x + 0.6141
2D-MS	70%	0.952	18.54	49.17	y = 0.7437x—8.1637
	75%	0.952	17.02	45.12	y = 0.7968x—8.7468
	80%	0.952	15.53	41.16	y = 0.85x—9.3299
	85%	0.952	14.08	37.32	y = 0.9031x—9.913
	90%	0.952	12.68	33.63	y = 0.9562x—10.496
	95%	0.952	11.37	30.16	y = 1.0093x—11.079
	**100%**	**0.952**	**10.17**	**26.97**	**y = 1.0624x—11.662**
3D-RGB	70%	0.9399	9.49	25.62	y = 0.9282x—6.1406
	75%	0.9399	7.69	20.78	y = 0.9945x—6.5793
	80%	0.9399	6.20	16.76	y = 1.0608x—7.0179
	85%	0.9399	5.28	14.26	y = 1.1271x—7.4565
	**90%**	**0.9399**	**5.23**	**14.12**	**y = 1.1934x—7.8951**
	95%	0.9399	6.07	16.40	y = 1.2597x—8.3337
	100%	0.9399	7.52	20.30	y = 1.326x—8.7723

*PH* plant height, *R*^*2*^ coefficient of determination, *RMSE* root-mean-square error, *NRMSE* normalized root-mean-square error. The best result in terms of R^2^, RMSE and NRMSE values were boldfaced

As presented in Table 1, the R^2^ between UAV measurement and ground measurement did not increase. However, calibration did significantly reduce the RMSE and NRMSE. In 2D-RGB, 80% of plant height was the most optimum result, with the R^2^ of 0.9915, RMSE of 1.44 cm, NRMSE of 5.02%, and the fitting equation was y = 0.9796x + 0.4909. In 2D-MS, 100% plant height was the most optimum result, with R^2^ of 0.952, RMSE of 10.17 cm, NRMSE of 26.97%, and the fitting equation was y = 1.0624x − 11.662. In 3D-RGB, 90% of plant height was the most optimum result, with R^2^ of 0.9399, RMSE of 5.23 cm, NRMSE of 14.12%, and the fitting equation was y = 1.1934x − 7.8951. In this study, from the comparison of these three optimal results, 80% of plant height values extracted from 2D-RGB UAV imagery was the most suitable for monitoring plant height during the whole growth period of faba bean.

### Comparison of the most suitable plant height and ground measurement

**Fig. 3:**
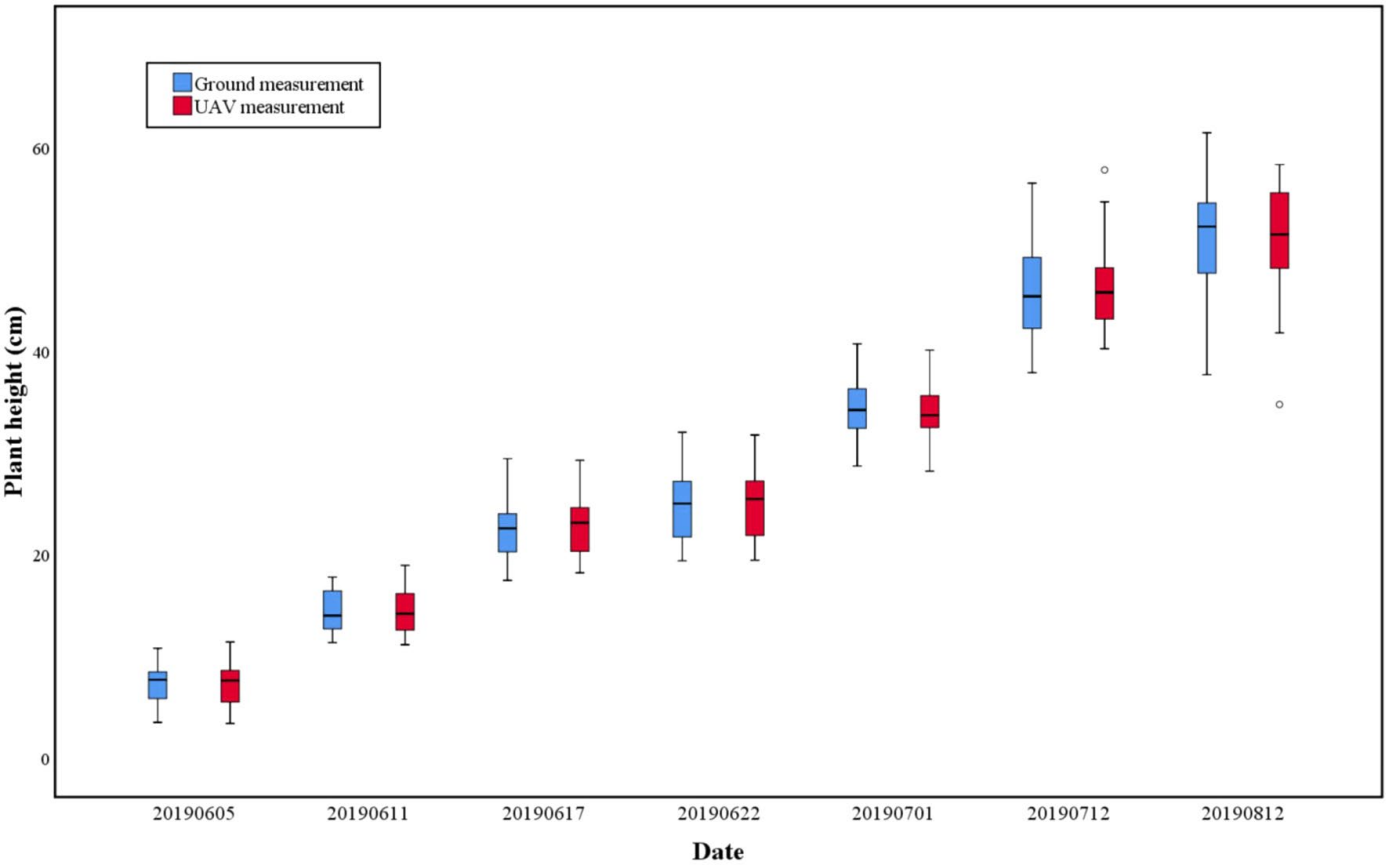
Comparison of plant height between ground measurement and UAV measurement

The 80% of plant height values extracted from 2D-RGB UAV imagery was compared with the ground measured values, which was represented by box-plot (Fig. 3).

As Fig. 3 showed, during the whole growth period of faba bean, the average plant height extracted from UAV imagery was almost consistent with that of the ground measured. Meanwhile, the dynamic changes of plant height were well represented by UAV measured. This implied that the 2D-RGB UAV imagery was effective in estimating the plant height in *V. faba*.

### Correlation analysis between plant height and yield in faba bean

**Fig. 4:**
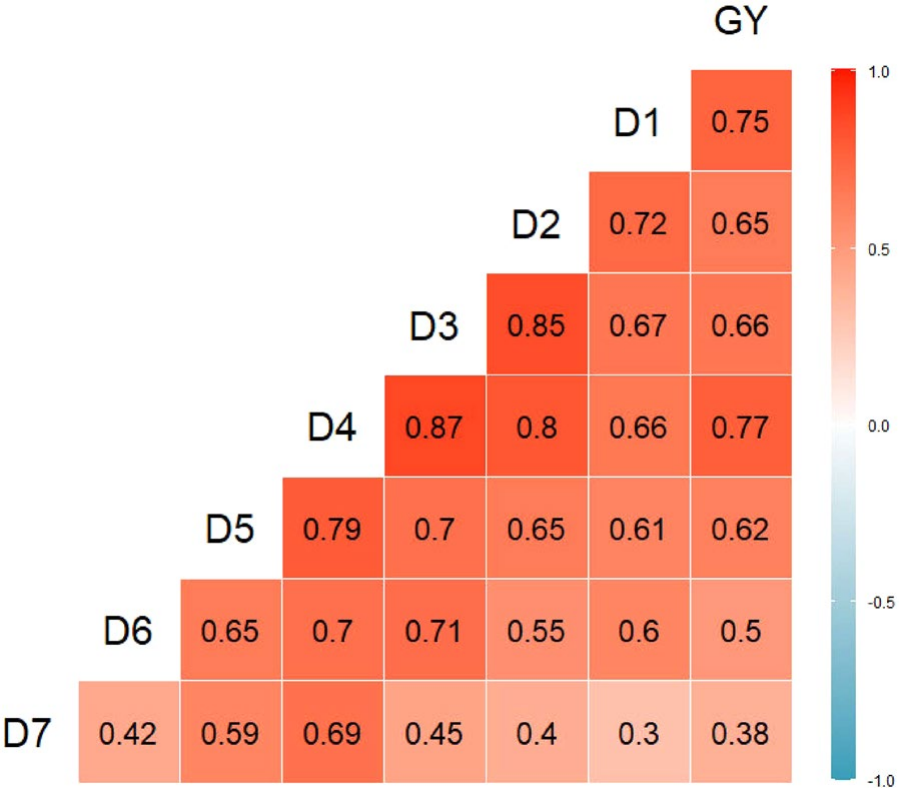
Correlation map between yield and plant height in different time. GY: Grain yield; D1: Date 1 (20,190,605); D2: Date 2 (20,190,611); D3: Date 3 (20,190,617); D4: Date 4 (20,190,622); D5: Date 5 (20,190,701); D6: Date 6 (20,190,712); D7: Date 7 (20,190,812)

Pearson's correlation coefficients were initially estimated to verify the association between yield and the optimal plant height values at seven time points, and the results were shown in Fig. 4.

As presented in Fig. 4 that there was a strong correlation between plant height and yield at seven time points, ranged from 0.3 to 0.87. Among them, D4 (20190622) had the highest correlation with yield, reaching 0.77, and D7 (20190812) had the lowest correlation with yield, only 0.38. Through the comparisons, it was found that the correlation coefficients between the two adjacent time points were higher than those between the cross-time points, among which the correlation coefficients between D3 (20190617) and D4 (20190622) were the highest (0.87), while the correlation coefficient between D1 (20190605) and D7 (20190812) was the lowest (0.3).

### Construction and validation of yield estimation model for faba bean

The plant height data at different time points and combinations of different time points were selected to estimate the yield of faba bean by using SVM, RF and DT machine learning algorithms. The results were shown in Additional file 1: Table S1. In the SVM-based algorithm, the range of R^2^, RMSE and NRMSE was 0.1441 ~ 0.7238, 823.54 ~ 1387.26 kg ha^−1^ and 18.38% ~ 30.96%, respectively. The best estimation result was based on D1 + D2 + D4 + D7 sample, and the obtained R^2^ was 0.7238, RMSE was 823.54 kg ha^−1^ and NRMSE was 18.38%. In the RF-based algorithm, the range of R^2^, RMSE and NRMSE was 0.1257 ~ 0.6573, 877.06 ~ 1636.73 kg ha^−1^, and 19.57% ~ 36.53%, respectively. The best estimation result was based on D1 + D4 + D6 sample, and the obtained R^2^ was 0.6573, RMSE was 877.06 kg ha^−1^ and NRMSE was 19.57%. In the DT-based algorithm, the range of R^2^, RMSE and NRMSE was 0.1403 ~ 0.5971, 923.24 ~ 1368.67 kg ha^−1^ and 20.60% ~ 30.54%, respectively. The best estimation result was based on D1 + D4 sample and D1 + D4 + D6 sample, and the obtained R^2^ was 0.5971, RMSE was 923.24 kg ha^−1^ and NRMSE was 20.60%.

**Fig. 5:**
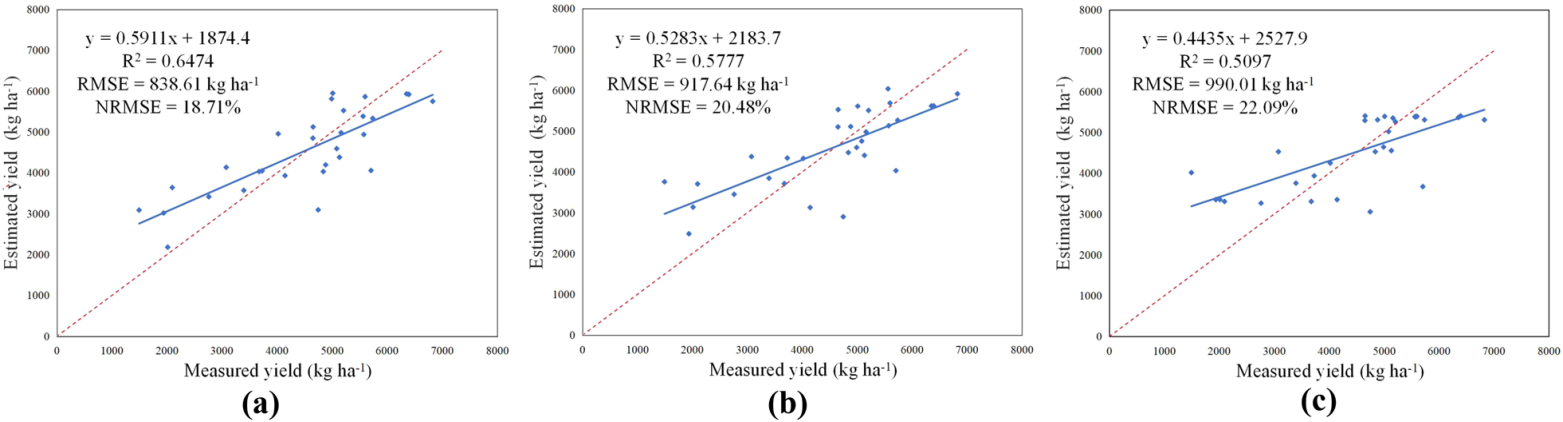
Comparison of estimated yield and measured yield by machine learning algorithms. **a** Support Vector Machines; **b** Random Forests; **c** Decision Trees

The estimated yield based on three machine learning algorithms were compared with measured yield, and the results were shown in the 1:1 line diagram of measured and estimated values (Fig. 5). It could be found that the estimated values of faba bean yield based on plant height was in good agreement with the measured values. The R^2^ between the estimated and measured yield based on SVM algorithm was 0.6474, RMSE was 838.61 kg ha^−1^ and NRMSE was 18.71%. The R^2^ between the estimated yield and measured yield based on RF algorithm was 0.5777, RMSE was 917.64 kg ha^−1^ and NRMSE was 20.48%. The R^2^ between the estimated yield and measured yield based on DT algorithm was 0.5097, RMSE was 990.01 kg ha^−1^ and NRMSE was 22.09%.

### The yield estimation in different number of time points

By comparing the estimation results of the three machine learning algorithms for faba bean yield under different number of time points (Table 2), it can be found that in the SVM-based algorithm, when the number of time points was 7, the average R^2^ was the highest (0.5776) and the corresponding RMSE, NRMSE was 981.26 kg ha^−1^, 21.90%, respectively. In the RF-based algorithm, when the number of time points was 5, the average R^2^ was the highest (0.5004) and the corresponding RMSE, NRMSE was 990.55 kg ha^−1^, 22.11%, respectively. In the DT-based algorithm, when the number of time points was 4, the average R^2^ was 0.5004 and the corresponding RMSE, NRMSE was 1026.93 kg ha^−1^, 22.92%, respectively. Therefore, in the study of faba bean yield estimation based on different machine learning algorithms, the appropriate number of time points should be selected to obtain the best estimation results.

**Table 2:** Results of yield estimation in different number of time points

Number of Time Points	SVM			RF			DT		
	R^2^	RMSE (kg ha^−1^)	NRMSE (%)	R^2^	RMSE (kg ha^−1^)	NRMSE (%)	R^2^	RMSE (kg ha^−1^)	NRMSE (%)
1	0.3296	1261.56	28.15	0.3683	1222.19	27.28	0.4018	1144.99	25.55
2	0.3873	1175.28	26.23	0.4453	1092.13	24.37	0.4629	1068.64	23.85
3	0.4785	1100.34	24.56	0.4877	1029.59	22.98	0.4937	1036.40	23.13
4	0.5236	1052.76	23.49	0.4951	1003.83	22.40	0.5004	1026.93	22.92
5	0.5512	994.17	22.71	0.4989	990.55	22.11	0.4975	1027.21	22.92
6	0.5744	958.9	21.40	0.4955	984.11	21.96	0.4912	1031.83	23.03
7	0.5776	981.26	21.90	0.4710	1008.70	22.51	0.4815	1038.23	23.17

## Discussion

### Extraction of imaged-based plant height feature in faba bean

In recent years, the research of extracting crop plant height based on UAV remote sensing data has been widely used, and its applicability and accuracy have also been recognized by a large number of agricultural researchers. Lidar is an active sensor, which uses the laser pulse in the 600 ~ 1000 nm region to determine the distance to the object [46]. Compared with other sensors, laser radar is less affected by environmental conditions and has relatively high estimation accuracy for several crop phenotyping traits [47]. However, the price of lidar is generally high. In contrast, the RGB camera is affordable and easy to operate. Therefore, many researchers tend to use consumer-level sensors, but most of the previous studies were based on a single sensor data to extract plant height [30, 32]. In this study, 2D-RGB, 2D-MS and 3D-RGB UAV imagery were collected respectively based on RGB camera and multispectral sensors, and it was found that the average plant height values of each plot extracted from these UAV imagery was lower than the ground measured values, which was consistent with the previous studies [48, 49]. In view of the fact faba bean has branches, easy to lodging, and the plant height performance of each branch is quite different. Therefore, the maximum plant height of each plot and the percentage were used to compare and analyze with the ground measured values in this study. It was found that 80% of plant height extracted from 2D-RGB images was the most ideal for estimating the plant height, indicating that the plant height extracted by this method can be reasonably used to estimate the actual plant height of faba bean. In addition, by comparing these three types of data, it was found that the accuracy of plant height data was determined by the image resolution. Some previous studies have shown that high resolution images can improve the accuracy of the plant height model [50, 51].

### Yield estimation using UAV measurement of plant height in faba bean

Plant height is one of the important agronomic traits in crop research, which can reflect crop growth status and is closely related to yield [52]. Yin et al. [53] took corn as the research object, collected plant height data from 2008 to 2010 at three time points V6, V10 and V12 respectively, and estimated annual corn yield based on the collected data. The obtained R^2^ range was 0.32–0.87, among them, V10 (R^2^ ≥ 0.54) and V12 (R^2^ ≥ 0.69) were considered to be able to estimate the corn yield. Other prior studies have addressed the importance of plant height in yield estimation [54, 55], but most of them were based on single time point plant height data to estimate yield and analyze the accuracy of its estimation. In this study, the yield of faba bean was estimated not only based on single time point plant height data, but also based on multiple time points combination of plant height data. The results showed that the single time point plant height could be used for estimation of faba bean yield, but its estimation effect was poor. When estimating faba bean yield based on multiple time points plant height, it was found that the estimation effect would be significantly improved, and the R^2^ of the best estimation model increased by 0.1252, RMSE and NRMSE decreased 126.83 kg ha^−1^ and 2.83%, respectively. The results also showed that the correlation coefficients between the plant height and yield at early growth stage (D1, D2) and middle growth stage (D3, D4, D5) were higher than the late growth stage (D6, D7) in this study. It could be attributed to the fact that the plant main stem height at the late growth stage tended to be stable, but the yield was still in the accumulation process, leading to its estimates of the effect was not ideal. Therefore, in the process of estimating crop yield based on remote sensing data, the selection of model independent variables was crucial. In this study, only one year plant height data was used to construct the yield model. In the future, it is necessary to further analyze and use the data of multiple years and multiple locations to obtain a more universal yield estimation model of faba bean.

### Performance of machine learning algorithms in faba bean yield estimation

Machine learning algorithms has been widely used in crop yield estimation research, and the methods used in this study were in accordance with the previous applications where the machine learning algorithms were successfully applied and conducted [56]. In this study, three machine learning algorithms of SVM, RF and DT were respectively used to estimate the yield of faba bean. By comparing the estimation results generated using three machine learning algorithms (Additional file 1: Table S1), it could be found that the yield estimation effect of SVM was the best, followed by RF and DT. Guo et al. [57] found that SVM algorithm can improve the accuracy of crop yield estimation, which is consistent with the results of this study. RF method is suitable for large data sets and maintains high accuracy, but it usually leads to over-fitting phenomenon, resulting in slightly worse prediction results [58]. DT has poor predictive performance for data with time sequence or large data sets [59], which can explain its unsatisfactory estimation result of faba bean yield in this study. Multiple types of remote sensing data and advanced machine learning algorithms can be used to estimate the yield of faba bean in the future.

## Conclusions

In the present study, three types of UAV imagery were used to extract the plant height of faba bean. The most optimum plant height values were used to estimate the yield of faba bean by using machine learning algorithms (SVM, RF and DT). Overall, the results of this study presented that UAV imagery could provide accurate estimation of faba bean plant height and yield. This study will aid in finding a high-throughput and non-destructive way to estimate plant height and yield for faba bean, which would accelerate screening of germplasm and breeding materials.

## Methods

### Research area and test design

**Fig. 6:**
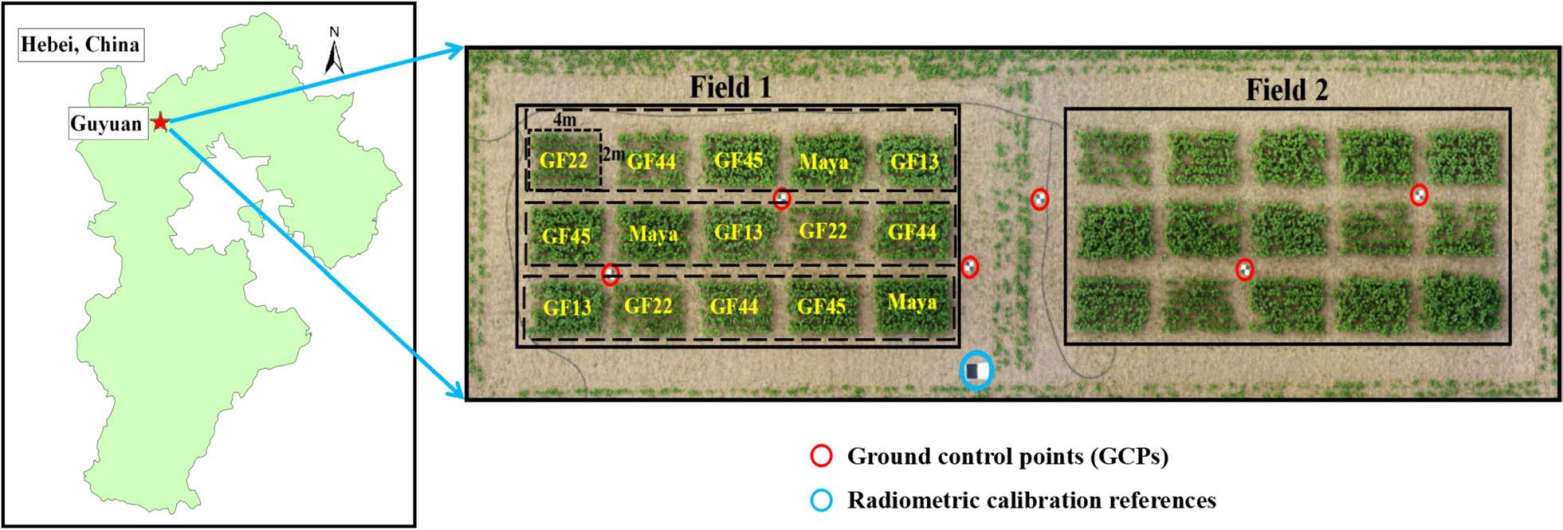
The geographical location and UAV sampling sites of the research area

The research area was located in Guyuan Experimental Station of Institute of Crop Sciences (ICS), Chinese Academy of Agricultural Sciences (CAAS), Zhangjiakou city (41° 14′ 33"—41° 56′ 55" N, 114° 50′ 38 "—116° 04′ 09" E, with average altitude 1,536 m) in Hebei Province, China. It belongs to temperate continental grassland climate, annual average temperature is 1.6℃, annual sunshine duration is 3,246 h, the shortest sunshine duration is 2,616 h, annual precipitation is 426 mm and the annual average frost-free period is 117 d. The geographical location and UAV sampling sites of the research area was shown in Fig. 6. The research area was divided into two experimental parts, each of them has five varieties, GF13, GF22, GF44, GF45 and Maya. Three replicates were used with completely randomized trial design and each plot area was 4 m × 2 m = 8 m^2^. All cultivars were planted on April 18, 2019 at a depth of 5 ~ 8 cm. 40 seeds were planted in each row, and six rows in each plot. After emergence, manual weed extraction were conducted as needed, and no fertilizers were used during this study. In order to improve the accuracy of image stitching and plant height extraction, six ground control points (GCPs) were located in the field for later geometric correction and image registration.

### Acquisition of ground data

The ground data collected in this study included plant height and yield, and the acquisition time of plant height was consistent with UAV flight date, the acquisition time of yield was August 22, 2019. Ground-based plant height was obtained as follows: 12 representative plants were randomly selected in each plot. The ruler was used to measure the distance from the top to the ground of each plant under the natural state. And the average value of 12 plants was taken as the ground measured plant height for each plot. Ground-based yield was obtained as follows: all plants in each plot were harvested and dry grains were weighed as the yield for each plot.

### Acquisition and processing of UAV remote sensing data

**Fig. 7:**
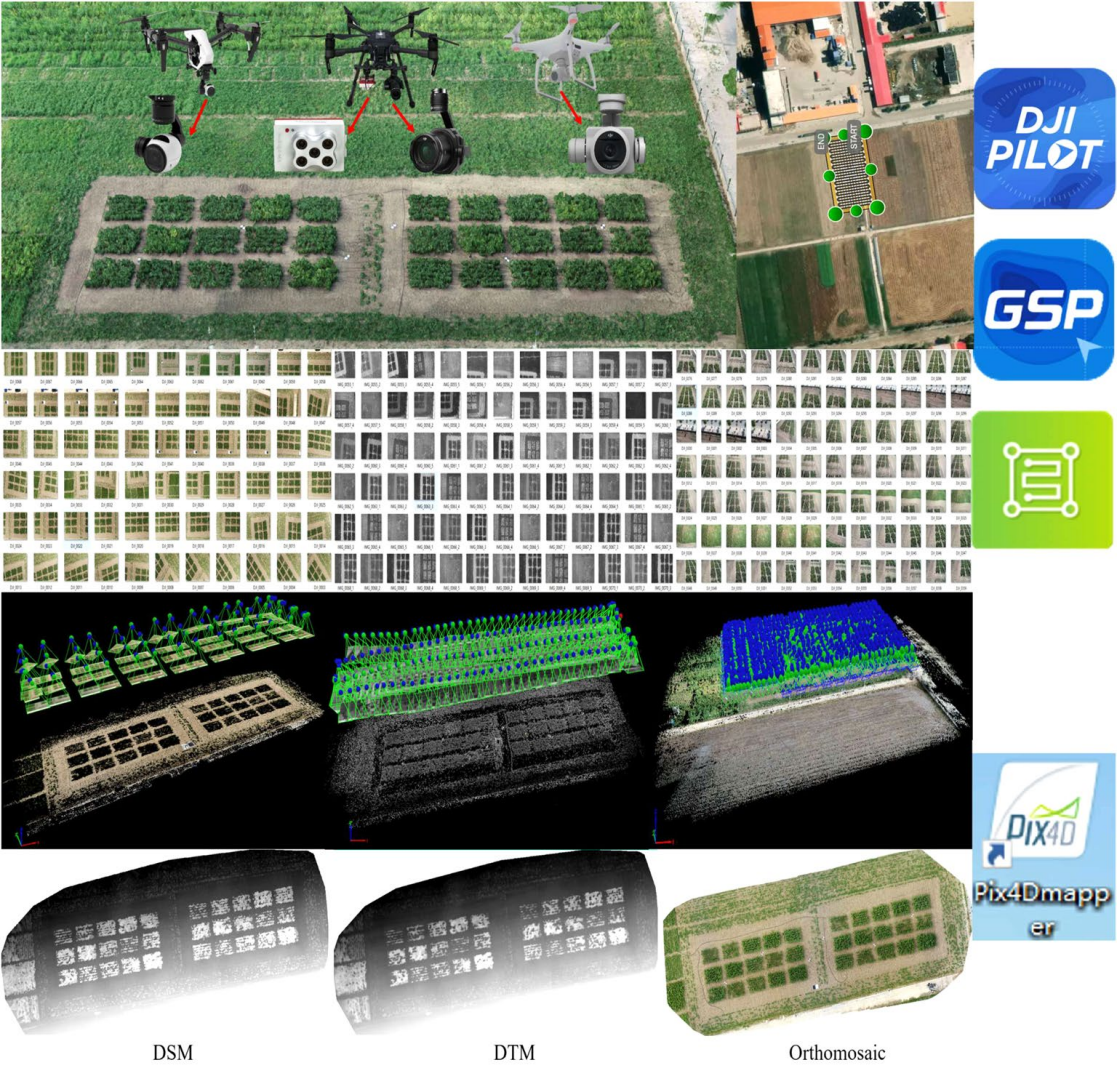
Acquisition and processing of UAV remote sensing data

The acquisition and processing of UAV remote sensing data (Fig. 7) in this study mainly includes two stages: (1) Acquired UAV imagery based on flight planning software (DJI GS Pro, DJI Pilot, Pix4Dcapture); (2) Mosaiced UAV imagery in the structure from motion (SfM)-based software (Pix4DMapper) and then automatically generated digital surface model (DSM), digital terrain model (DTM) and Orthomosaic.

DJI Inspire 1 (SZ DJI Technology Co., Shenzhen, China), DJI Matrice 210 (SZ DJI Technology Co., Shenzhen, China) and DJI Phantom 4 (SZ DJI Technology Co., Shenzhen, China) were used to acquire UAV images of the experimental plots. The detailed parameters of three UAVs were shown in Table 3.

**Table 3:** Detailed parameters of three UAVs

Items	DJI inspire 1	DJI matrice 210	DJI phantom 4
Size/mm	438 × 451 × 301	883 × 886 × 398	196 × 289.5 × 289.5
Body weight/g	2935	4800	1380
Wheelbase/mm	581	643	350
Endurance/min	18	24	28
Max speed/(m/s)	22	17	20
Flight planning software	DJI GS Pro	DJI Pilot	Pix4Dcapture

This study collected three types of UAV image data: 2D-RGB, 2D-MS and 3D-RGB. Zenmuse X3 camera carried by DJI Inspire 1 and Zenmuse X7 camera carried by DJI Matrice 210 were used to collect 2D-RGB images. RedEdge-MX sensor carried by DJI Matrice 210 was used to collect 2D-MS images. Phantom camera carried by DJI Phantom 4 was used to collect 3D-RGB images. The size of Zenmuse X3 camera is 1/2.3" and the effective pixel is 12 million. The size of Zenmuse X7 camera is 151 mm × 108 mm × 132 mm and the effective pixel is 24 million. The size of Phantom camera is 1/2.3" and the effective pixel is 12.4 million. The RedEdge-MX sensor has a weight of 232 g and a size of 87 mm × 59 mm × 45.4 mm. It contains five bands: blue (475 nm), green (560 nm), red (670 nm), red-edge (720 nm) and near-infrared (840 nm), with the resolution of 1280 × 960.

The flights planning (Table 4) were applied under the conditions of cloudless and low wind speed.

**Table 4:** Flights planning parameters for UAV imagery system

Flight data	Type	Altitude	Forward overlap	Side overlap	UAV	Sensor
2019/6/5	2D-RGB	25 m	85%	80%	DJI Matrice 210	Zenmuse X7
2019/6/11	2D-RGB	25 m	85%	80%	DJI Matrice 210	Zenmuse X7
2019/6/17	2D-RGB	25 m	85%	80%	DJI Matrice 210	Zenmuse X7
2019/6/22	2D-RGB	10 m	85%	80%	DJI Inspire 1	Zenmuse X3
2019/7/1	2D-RGB	10 m	85%	80%	DJI Inspire 1	Zenmuse X3
2019/7/12	2D-RGB	25 m	85%	80%	DJI Matrice 210	Zenmuse X7
2019/8/12	2D-RGB	25 m	85%	80%	DJI Matrice 210	Zenmuse X7
2019/6/11	2D-MS	25 m	80%	75%	DJI Matrice 210	RedEdge-MX
2019/6/18	2D-MS	25 m	80%	75%	DJI Matrice 210	RedEdge-MX
2019/7/12	2D-MS	25 m	80%	75%	DJI Matrice 210	RedEdge-MX
2019/8/6	2D-MS	25 m	80%	75%	DJI Matrice 210	RedEdge-MX
2019/8/12	2D-MS	25 m	80%	75%	DJI Matrice 210	RedEdge-MX
2019/6/10	3D-RGB	10 m	90%	85%	DJI Phantom 4	Phantom camera
2019/6/23	3D-RGB	10 m	90%	85%	DJI Phantom 4	Phantom camera
2019/6/30	3D-RGB	10 m	90%	85%	DJI Phantom 4	Phantom camera
2019/7/11	3D-RGB	10 m	90%	85%	DJI Phantom 4	Phantom camera
2019/7/30	3D-RGB	10 m	90%	85%	DJI Phantom 4	Phantom camera
2019/8/12	3D-RGB	10 m	90%	85%	DJI Phantom 4	Phantom camera

The Pix4DMapper 4.4.12 software (Pix4D SA, Lausanne, Switzerland) [60, 61] was used to optimize the interior and exterior parameters of the images. A sparse dense cloud based on the structure-from-motion (SfM) technique and point clouds based on the multi-view stereo (MVS) with multiple control points collected were used. For 2D-RGB and 3D-RGB images, we imported the images into Pix4Dmapper, which would automatically read the position and orientation system (POS) data and the camera configuration information. Then, we set the number of matching feature points and the type of output images in the process of stitching. Finally, the DSM, DTM and Orthomosaic were outputted in different files. For 2D-MS images, its processing steps were consistent with RGB images. Moreover, the stitching of multi-spectral images needs to use the calibrated reflectance panel image taken before UAV takeoff to carry out reflectance calibration.

### Extraction of plant height

The DSM and DTM output files from Pix4DMapper software were used in Equation One to obtain crop surface model (CSM). Then CSM was used to extract the plant height data in each plot by using the ROI tool in the ENVI 5.3 software (ITT Visual Information Solutions, Boulder, CO, USA) [62], and the maximum plant height of each plot was selected for subsequent data processing. The calculation equation for Equation One was as follows:

1 $$CSM = DSM - DTM$$

### Machine learning algorithms

SVM [63] is a widely used machine learning method for classification and regression analysis, which is based on the key concepts of statistical learning theory. The basic SVM model is a linear classifier, and the maximum interval is defined in the feature space. The maximum interval refers to those sample points closest or furthest from the hyperplane in the two types of sample points, whether the sample is low-dimensional or high-dimensional. Since SVM can use kernel functions to convert highly nonlinear data into linearly separable data, so as to perform well on different data set. Overall, the SVM algorithm has great advantages in solving nonlinear, small sample and high dimensional problems. We conducted grid search based on radial basis kernel to determine the optimal values of hyperparameters C and γ, which influence the accuracy and generalisation capabilities of the SVM [64].

RF [65] could be regarded as a machine learning model that combines a large number of regression trees. In regression modeling tasks, the main advantages of random forests are to minimize the risk of overfitting. At the same time, the importance of all predictive variables could be divided to evaluate the contribution of each predictive variable to the model, and then eliminate redundant variables. The key step to construct RF is to split the regression tree and take the average value of all trees as the prediction result of the final output. As Yang et al. [66] set in the research, we defined and optimized two hyperparameters in the random forest algorithm: one is the number of trees (ntree is 500), the other is the number of different variables for tree node splitting (mtry is one-third of the total number of variables).

DT [67] is a tree structure applied to classification and regression. A decision tree contains one root node, several internal nodes and several leaf nodes. The root node contains a full set of samples. Each internal node represents a test on an attribute, each branch represents a test output, and each leaf node represents a category. The decision-making process of the decision tree needs to start from the root node of the decision tree. The measured data are compared with the feature nodes of the decision tree, and the next comparison branch is selected according to the comparison results until the leaf node is used as the final decision result to complete the regression. We combined cross validation and grid search to select hyperparameters of DT.

### Statistical analysis

Pearson correlation and machine learning algorithms (SVM, RF, DT) were calculated to check relationship and estimation between the plant height and yield, which were completed by RStudio 4.0.2 (RStudio, Inc. Boston, USA). In cross validation, the plant height values of a single time point or a combination of multiple time points were randomly divided into five parts. Four-fifths of the samples (n = 24, plant height or yield of faba bean) were randomly selected as the modeling data set, and the other one-fifths of the samples (n = 6, plant height or yield of faba bean) were used as the validation data set. The yield estimation model was verified by the sample data of validation set, and the final results were shown by the 1:1 plot of measured and estimated values.

To evaluate the model performance, three evaluation indicators were used to determine the accuracy of the yield estimation, namely the coefficient of determination (R^2^), root-mean-square error (RMSE), and normalized root-mean-square error (NRMSE) [48]. The calculation equations for these parameters are given as follows:

2 $$R^{2} = 1 - \sum\nolimits_{i = 1}^{n} {\left( {x_{i} - y_{i} } \right)^{2} } /\sum\nolimits_{i = 1}^{n} {\left( {x_{i} - \overline{x} } \right)^{2} }$$

3 $$RMSE = \sqrt {\sum\nolimits_{i = 1}^{n} {\left( {y_{i} - x_{i} } \right)^{2} } /n}$$

4 $$NRMSE = RMSE/\overline{x}$$

Where x_i_ is plant height or plant yield of faba bean, ${\overline{\text{x}}}$ is the average plant height or yield, y_i_ is the plant height or yield predicted by the model, and n is the number of data points.

## Supplementary Information

**Additional file 1: Table S1**. Results of yield estimation by machine learning algorithms.

## Data Availability

The datasets used in this study is available from the corresponding author on reasonable request.

## Abbreviations

2D-RGB: Two-dimensional red–green–blue
2D-MS: Two-dimensional multispectral
3D-RGB: Three-dimensional red–green–blue
UAV: Unmanned aerial vehicle
SVM: Support vector machines
RF: Random forests
DT: Decision trees
R^2^: Coefficient of determination
RMSE: Root-mean-square error
NRMSE: Normalized root-mean-square error
ICS: Institute of Crop Sciences
CAAS: Chinese Academy of Agricultural Sciences
GCPs: Ground control points
SfM: Structure from motion
DSM: Digital surface model
DTM: Digital terrain model
CSM: Crop surface model
